# A framework for tracing timber following the Ukraine invasion

**DOI:** 10.1038/s41477-024-01648-5

**Published:** 2024-03-11

**Authors:** Thomas Mortier, Jakub Truszkowski, Marigold Norman, Markus Boner, Bogdan Buliga, Caspar Chater, Henry Jennings, Jade Saunders, Rosie Sibley, Alexandre Antonelli, Willem Waegeman, Victor Deklerck

**Affiliations:** 1 World Forest ID, Washington, DC USA; 2 Department Data Analysis and Mathematical Modelling, Ghent University, Ghent, Belgium; 3 Department of Biological and Environmental Sciences, University of Gothenburg, Gothenburg, Sweden; 4 Gothenburg Global Biodiversity Centre, Gothenburg, Sweden; 5 Agroisolab GmbH, Juelich, Germany; 6 Preferred by Nature, Ho Chi Minh, Vietnam; 7 University Stefan cel Mare Suceava, Suceava, Romania; 8 Royal Botanic Gardens, Kew, Richmond, UK; 9 Plants, Photosynthesis, and Soil, School of Biosciences, University of Sheffield, Sheffield, UK; 10 Department of Biology, University of Oxford, Oxford, UK; 11 Meise Botanic Garden, Meise, Belgium

**Keywords:** Forestry, Stable isotope analysis, Forestry, Socioeconomic scenarios, Conservation biology

## Abstract

Scientific testing including stable isotope ratio analysis (SIRA) and trace element analysis (TEA) is critical for establishing plant origin, tackling deforestation and enforcing economic sanctions. Yet methods combining SIRA and TEA into robust models for origin verification and determination are lacking. Here we report a (1) large Eastern European timber reference database (*Betula*, *Fagus*, *Pinus*, *Quercus*) tailored to sanctioned products following the Ukraine invasion; (2) statistical test to verify samples against a claimed origin; (3) probabilistic model of SIRA, TEA and genus distribution data, using Gaussian processes, to determine timber harvest location. Our verification method rejects 40–60% of simulated false claims, depending on the spatial scale of the claim, and maintains a low probability of rejecting correct origin claims. Our determination method predicts harvest location within 180 to 230 km of true location. Our results showcase the power of combining data types with probabilistic modelling to identify and scrutinize timber harvest location claims.

## Abstract (web summary)

Timber harvest claims can be scrutinized based on a combination of wood chemical composition and species distribution. The location of timber harvest can be determined within 180 to 230 km of true location across Eastern Europe.

## Main

Russia’s invasion of Ukraine sparked global responses designed to penalize Russia and thwart continuing aggression. The UK and the European Union announced economic sanctions packages, including a ban on the direct imports of wood products from Russia and Belarus^1,2^. The USA increased tariffs on wood imports from both countries (https://hts.usitc.gov). These interventions, combined with bans by the Forest Stewardship Council and the Programme for the Endorsement of Forest Certification^3,4^, transformed timber products harvested in Russia and Belarus into ‘conflict timber’ in western markets^5^.

Companies operating in the UK, European Union and USA have long relied on timber imports from Russia and Belarus, particularly birch (*Betula* spp.), for construction^6^. By weight, 12% of all European Union 2021 wood product imports under Chapter 44 of the Harmonized Tariff Schedule were imported from Russia and Belarus (https://ec.europa.eu/eurostat/comext/newxtweb/). While there is emerging evidence of companies seeking replacement markets, demand for birch, beech (*Fagus* spp.), pine (*Pinus* spp.) and oak (*Quercus* spp.) products remains high^6^. As a result, there has been a rise in trade through secondary markets, suggesting efforts to disguise origin (location of harvest) to evade sanctions or tariffs^6^.

Origin misdeclaration undermines the policy intent of sanctions/tariffs but also violates existing environmental laws, including the European Union Timber Regulation and UK Timber Regulation^7,8^. Enforcement officials implementing both timber import regulations and sanctions need scientific tools to interrogate location of harvest claims (national, sub-national or even concession level). Checking timber harvest location claims can be done in two ways: (1) verification, an assessment based on reasonable doubt over the claimed origin (for example, ‘Can this wood sample originate from this location?’) or (2) determination, where the harvest location is predicted without taking into account a priori information (‘Where does this wood sample originate from?’)^9^. For verification, a robust dataset from the claimed origin is needed, while for determination, a much larger geographic range and data set are required.

One of the most widely used scientific techniques for origin determination, stable isotope ratio analysis (SIRA), measures ratios of naturally occurring stable isotopes which vary predictably across space, in correlation with environmental conditions^10–13^. Although SIRA has been successful in origin prediction, its wider applicability is limited by the lack of (1) extensive reference data and (2) resolving power at small spatial scales^14^. Trace element analysis (TEA) has been proposed as an alternative^15^, as the trace element composition of forest biomass reflects the bio-available and mobilized macro- and micro-nutrients present in soils^16^, providing a spatial signal to trace timber back to harvest location. Empirical studies performed in Africa and Borneo differentiated timber from country or cluster/site level origins based on TEA^16,17^.

Studies using SIRA or TEA to address timber origin queries have framed the determination problem as a classification task^15,16,18^, wherein the objective is predicting the origin of a sample from a finite set of defined locations. While this approach may yield satisfactory results in scenarios with limited spatial range, it introduces several challenges once the range of interest expands. These include increasing complexity of the classification problem and a potential loss in predictive accuracy due to arbitrarily defined populations and unaccounted spatial structure. Creating a tool for real-world application, which can address changing rates of deforestation, illegal logging or conflict across a region, necessitates models that are designed to adapt to new scales and enforcement challenges built on broad sampling methodologies, which capture changing signals across large continuous areas regardless of nation state borders or other arbitrarily defined locations.

**Fig. 1:**
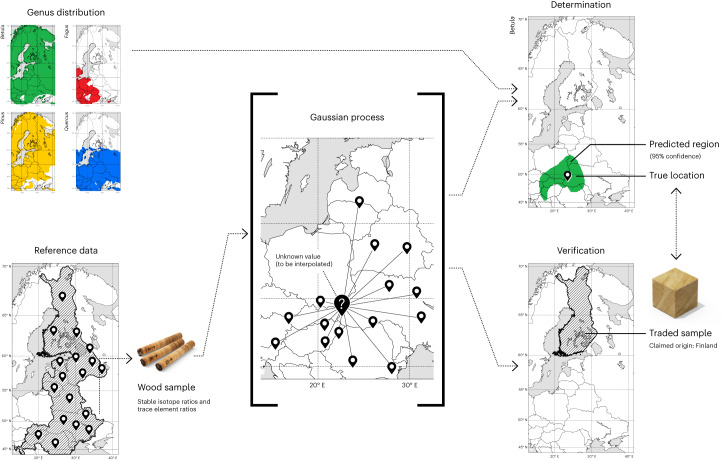
Flowchart illustrating the different steps within the framework. Top left: genus distribution included as a prior *p*(*x*) in the model. Bottom left: reference data as SIR and TE concentrations (*y*) from wood samples in locations (*x*). Middle: GP calculating the SIR and TE concentrations in each location in the grid (*p*(*y* ∣ *x*)). Top right: Bayesian determination model for harvest location. Bottom right: verification statistical test to discredit country claims.

We therefore propose a framework for the verification and determination of harvest location based on Gaussian process (GP) regression. GP regression models use values in sampled locations to estimate values in surrounding locations^19^, allowing us to consider a large continuous area. GP models estimate the covariance between measurements as a function of the distance between locations at which they were taken. This allows deriving the probability of observing a specific measurement at any location in the study area, in a way that accounts for varying levels of prediction uncertainty. We use those probabilities to perform Bayesian inference of possible harvest locations. Our method extends our recent approach^20^ by both incorporating trace elements and allowing verification testing (see Fig. 1 for a general overview).

We apply this framework on a large-scale data set, consisting of 929 timber reference samples of 4 genera across 11 Eastern European countries (Table 1). Specifically, we (1) combine trace element (TE) and stable isotope ratio (SIR) data obtained from wood samples to perform verification and determination; (2) develop a statistical test to verify a sample against a claimed origin on different spatial scales, taking into account spatial dependency; (3) take a probabilistic approach to spatial modelling of SIR and TE data using GPs to determine harvest location; (4) incorporate prior information based on genus distribution; and (5) determine which SIR and TE are most important for determining location. The presented framework can be applied to both enforce sanctions/tariffs and to scrutinize harvest location claims under European Union Timber Regulation, UK Timber Regulation and the European Union Deforestation Regulation (EUDR).

**Table 1:** Country origins of the 929 reference samples used in this work

Country	Genus			
	*Betula*	*Fagus*	*Pinus*	*Quercus*
Belarus	20	0	0	10
Croatia	20	60	30	57
Estonia	50	0	0	10
Finland	30	0	36	0
Hungary	10	16	13	49
Latvia	56	0	0	4
Lithuania	0	0	65	34
Moldova	0	0	0	51
Romania	12	35	0	10
Russia	24	0	0	0
Slovakia	20	20	0	9
Ukraine	60	88	30	0

## Results

### Verification

Our statistical verification method combines SIR and TE data and takes into account every potential harvest location within an origin claim (country, region, plot…). For every potential location, we compute the normalized residuals of SIR and TE values observed in the test sample. We then perform a *χ*^2^ test on the sum of squared residuals and report the maximum of the *P* values for all locations; see Methods for details.

**Fig. 2:**
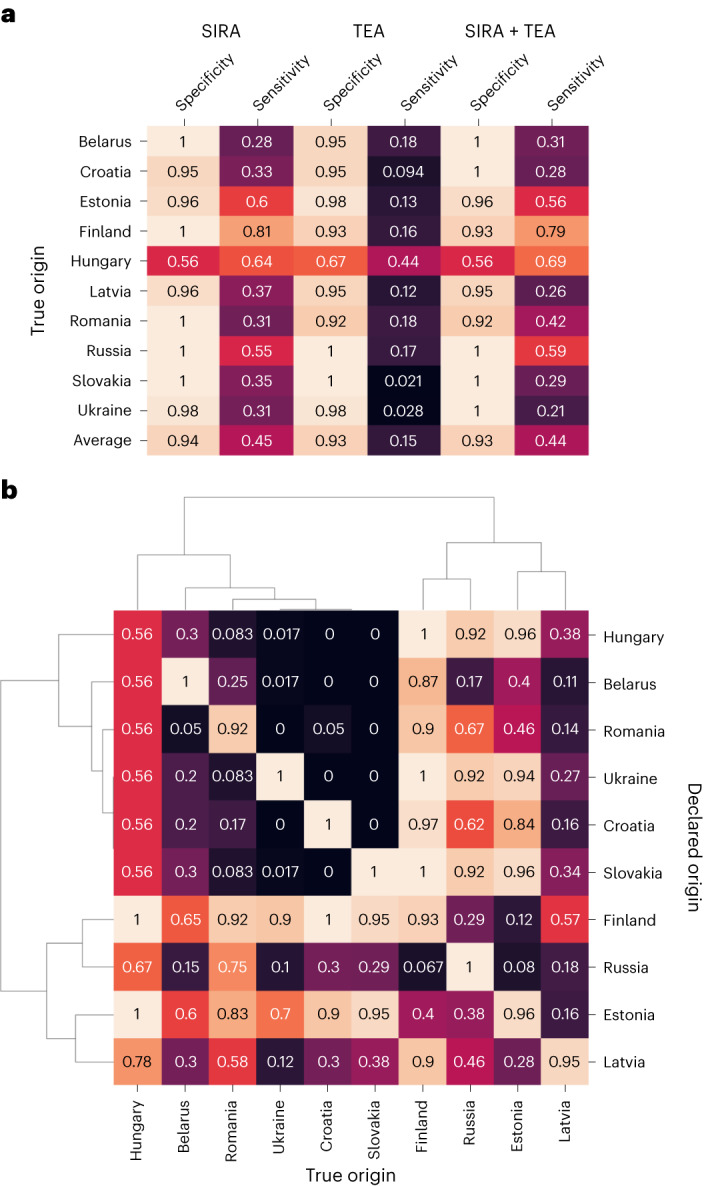
Verification test results. **a**, Accuracy of verification tests in *Betula* for countries in the study area using SIR, TE or both. Specificity is the fraction of samples with correct origin claims that were not rejected. Sensitivity is the fraction of samples with incorrect origin claims that were rejected. **b**, A clustered heat map of SIRA + TEA verification accuracies for all combinations of true (columns) and declared (rows) origins. The values on the main diagonal are specificities as the declared origin is correct. The values outside the main diagonal are sensitivities as the declared origin is incorrect. Average linkage clustering was performed using average of accuracies in both directions as the distance measure.

Figure 2a shows the accuracy of these models on test samples stratified by country, focusing on *Betula* as this genus is considered the main risk under conflict timber. The specificity (the probability of not rejecting a correct origin claim) is close to the intended level of 0.95 for all countries except Hungary. This may be due to the more limited sample numbers (10) obtained from Hungary. Sensitivity (the probability of rejecting an incorrect origin claim) is much lower for the TEA-based model than for models involving SIR data, possibly as a result of short autocorrelation ranges in many of the TEs.

The addition of TE data to the SIR-based model does not lead to an overall improvement in any of the metrics, in contrast to our determination method (see ‘Determination’). We observed large differences in sensitivities across origin countries and data types used, with the highest for samples from Finland analysed with SIRA (81%) and the lowest for samples from Slovakia analysed with TEA (2.1%). Fig. 2b shows the fraction of accurate responses achieved by the combined SIRA + TEA model for every combination of declared and true country of origin. The main diagonal shows specificities for every country, while the off-diagonal shows the sensitivities for specific misdeclaration scenarios. We see large differences in sensitivities between misdeclaration scenarios. Sensitivity tends to be low when samples are declared coming from a neighbouring country (for example, Ukraine declared as Romania) or when situated at similar latitudes (for example, Croatia declared as Ukraine).

Conversely, the verification test shows high sensitivity when a sample is misdeclared as coming from a distant country (for example, Russia declared as Slovakia) or when a large latitude difference exists between countries (for example, Finland declared as Latvia). Samples from Finland appear to be the most distinct from other locations, followed by Estonia and Russia. A hierarchical clustering analysis reveals two major clusters of countries that are clearly distinguishable from each other, with Finland, Estonia, Russia and Latvia forming one of the clusters, while the other consists of the remaining countries except Hungary as a possible outlier (Fig. 2b).

The sensitivity of the verification test increases when more precise harvest location claims are available (Section 1 in Supplementary Information), which will be a requirement under EUDR. The average sensitivity increases from 40% to 52% when level-1 administrative units are the declared harvest locations. For 0.25° × 0.25° concession harvest claims, the sensitivity increases to 60%. For samples from Russia, sensitivity increased from 59% at country level to 82% at concession level. This is accompanied by a slight decrease of specificity from 96% to 90%. The incorrect harvest locations claims were more likely to be rejected if they were made in areas where training samples were available to our model; when incorrect claims were simulated only within level-1 units where at least one sample has been collected, the sensitivity rose to 58% for level-1 declared locations and 67% for 0.25° × 0.25° concessions. This suggests that the accuracy of our verification test might improve with additional sampling.

### Determination

Our determination algorithm uses the trained GP regression models to compute the likelihood of observing the test sample at every location within the study area. We then use Bayes’ theorem to compute the probability of each possible location; see Methods for more details.

Table 2 shows the average and standard deviation of the great-circle distances between predicted and true origins, obtained after fourfold cross-validation on the reference data for each genus based on SIR data only, TE data only and a combination of the two data types (SIR + TE). Combining both data types leads to a considerable reduction in the average great-circle distance between actual location and the location deemed most likely by the model. The determination model for *Fagus* has the lowest average distance compared to the other determination models due to the smaller sampling area for this genus. For *Betula*, on average, the model predicts 228.64 km from the true timber harvest location, given SIR + TE data. TE-based models for *Betula*, *Quercus* and *Pinus* performed worse compared to the models based on SIRA alone.

**Table 2:** Average and standard deviation of great-circle distances (in kilometres) between predicted and true origins

Genus	SIRA	TEA	SIRA + TEA
*Betula*	349.31 (±25.46)	362.16 (±67.82)	228.64 (±15.49)
*Fagus*	296.55 (±14.74)	199.82 (±20.58)	179.48 (±19.88)
*Quercus*	274.91 (±25.53)	328.24 (±51.27)	211.03 (±43.15)
*Pinus*	266.45 (±42.03)	386.23 (±107.70)	216.50 (±54.16)

Values obtained after fourfold cross-validation on reference data for three different determination models: stable isotope ratio data only (SIRA), trace element concentration data only (TEA) and combination of both data types (SIRA + TEA).

**Fig. 3:**
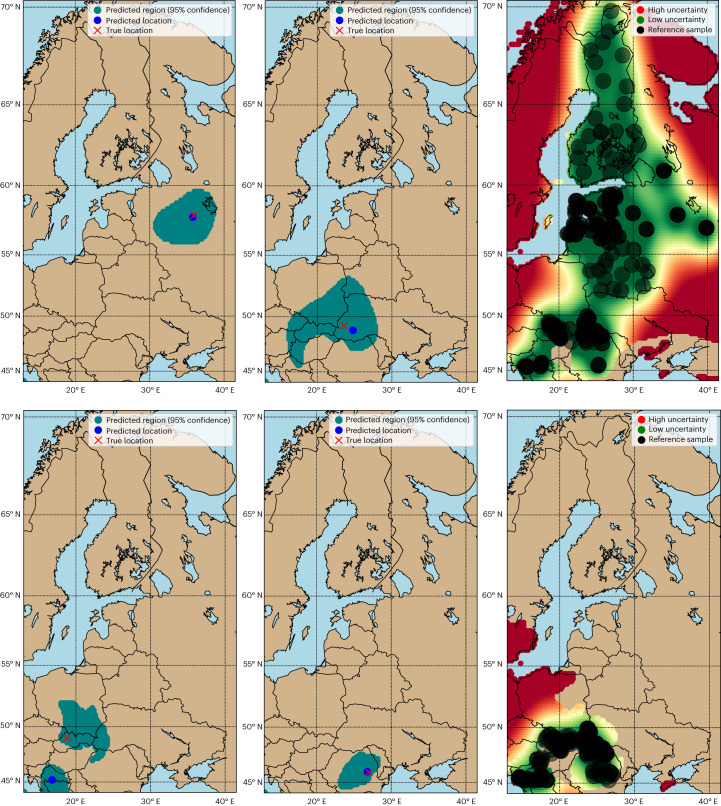
Determination for *Betula* and *Fagus*. Determination (left and middle) and uncertainty maps (right) for *Betula* on the first row and for *Fagus* on the last row. For the determination maps: 95% confidence regions are indicated in teal, the predicted timber harvest locations are indicated in blue, and the true locations of test samples are indicated by a red cross. For the uncertainty maps: reference samples in Table 1 are indicated by black circles, and low and high uncertainty locations are indicated by green and red, respectively. The 25th percentile of the uncertainty values serves as a cut-off for low uncertainty (green); values below are indicative of low uncertainty. Conversely, the 75th percentile of the uncertainty values acts as a cut-off for high uncertainty (red); values above are indicative of high uncertainty. The efficiency and coverage for *Betula* and *Fagus* are 333,006 km^2^, 92% and 230,274 km^2^, 90.91%, respectively (Section 2 in Supplementary Information).

**Fig. 4:**
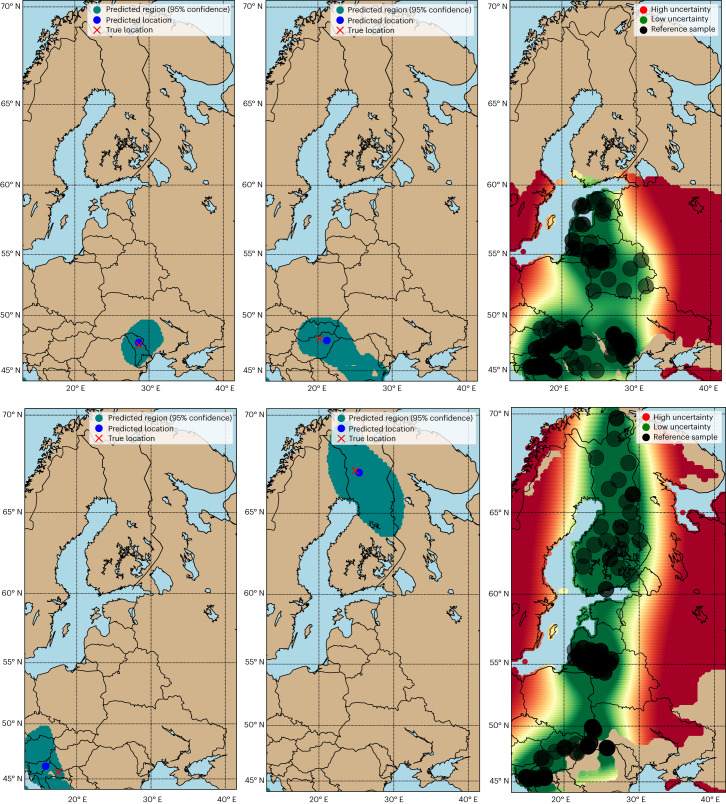
Determination for *Quercus* and *Pinus*. Determination (left and middle) and uncertainty maps (right) for *Quercus* on the first row and for *Pinus* on the last row. For the determination maps: 95% confidence regions are indicated in teal, the predicted timber harvest locations are indicated in blue, and the true locations of test samples are indicated by a red cross. For the uncertainty maps: reference samples in Table 1 are indicated by black circles, and low and high uncertainty locations are indicated by green and red, respectively. The 25th percentile of the uncertainty values serves as a cut-off for low uncertainty (green); values below are indicative of low uncertainty. Conversely, the 75th percentile of the uncertainty values acts as a cut-off for high uncertainty (red); values above are indicative of high uncertainty. The efficiency and coverage for *Quercus* and *Pinus* are 429,200 km^2^, 91.38% and 274,577 km^2^, 84.09%, respectively (Section 2 in Supplementary Information).

The accuracy of the determination model is visualized in the determination maps for *Betula*, *Fagus*, *Quercus* and *Pinus* (Figs. 3 and 4). For most cases, the predicted location (blue) is close to the true location of test samples (red cross). The confidence regions appear smaller for the determination model trained on the reference data for *Fagus* due to a smaller reference data sampling area for *Fagus* and smaller genus range inside the study area. In the right column, we present the uncertainty maps that show the reference data (black dots) utilized for training, alongside the uncertainty associated with each determination model. These visual aids are valuable for discerning areas where the determination model shows uncertainty and thus provide guidance for future data collection efforts. As demonstrated, Bayesian inference on top of GP regression allows for reliable and accurate determination.

**Fig. 5:**
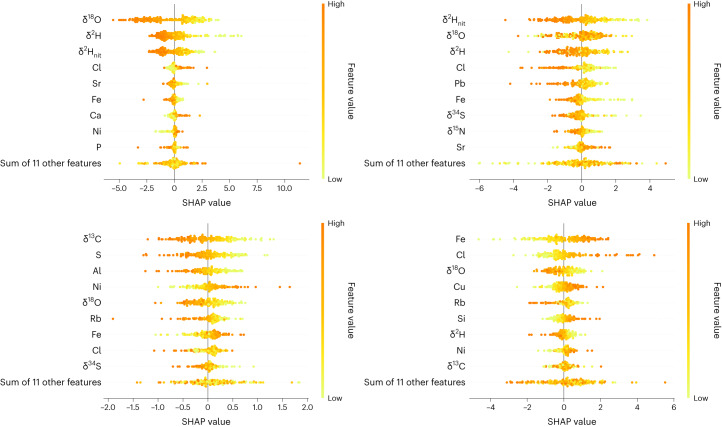
SHAP’s beeswarm plots for *Betula* and *Fagus*. Latitude (left) and longitude (right) for *Betula* on the first row and for *Fagus* on the last row. The feature value on the *y* axis corresponds to the measured value for a particular variable (SIR/TE). The SHAP value on the *x* axis corresponds to the impact on the model output. Variables on the *y* axis are sorted in decreasing order of average absolute SHAP value and represent the average impact across all test samples: the higher, the more important the variable in question.

**Fig. 6:**
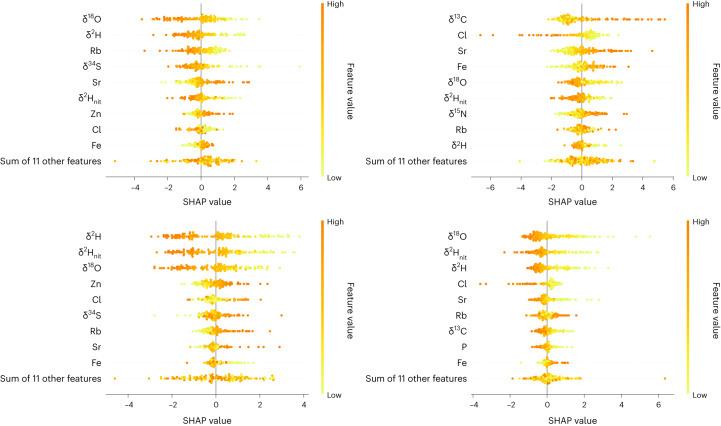
SHAP’s beeswarm plots for *Quercus* and *Pinus*. Latitude (left) and longitude (right) for *Quercus* on the first row and *Pinus* on the last row. The feature value on the *y* axis corresponds to the measured value for a particular variable (SIR/TE). The SHAP value on the *x* axis corresponds to the impact on the model output. Variables on the *y* axis are sorted in decreasing order of average absolute SHAP value and represent the average impact across all test samples: the higher, the more important the variable in question.

In Figs. 5 and 6 we present Shapley additive explanations (SHAP) beeswarm plots for latitude (left) and longitude (right) for *Betula*, *Fagus*, *Quercus* and *Pinus*. The feature value on the *y* axis corresponds to the measured value for a particular variable. The SHAP value on the *x* axis corresponds to the impact on the model output. A positive/negative SHAP value indicates an increasing/decreasing effect on the latitude or longitude prediction (that is, results in a higher/lower predicted longitude or latitude). Variables on the *y* axis are sorted in decreasing order of average absolute SHAP value and represents the average impact across all samples: the higher that value, the more important the variable in question. It is clear that both *δ*^2^H and *δ*^18^O have a large impact on latitude, given the determination model for *Betula*, *Quercus* and *Pinus*. In particular, isotope ratios are on average higher for timber samples that originate from the southern part of the study area. *δ*^13^C has a large impact on latitude for *Fagus* and longitude for *Quercus*. Our TEA models, including the SHAP beeswarm plots, indicate that chlorine (Cl), nitrogen (N), calcium (Ca), nickel (Ni), iron (Fe), lead (Pb), rubidium (Rb), silicon (Si), strontium (Sr) and zinc (Zn) play crucial roles in determining timber harvest location.

## Discussion

Current modelling practices for the use of SIRA and TEA to verify and determine timber harvest location are limited in statistical power, spatial resolution, number of data types considered or number of reference samples included in the model. Our framework allows timber harvest verification and determination by means of spatial modelling of stable isotope and trace element data via GPs, while taking into account genus distribution. GP models have been used by others to derive variance estimates for origin determination in animals^21,22^; however, in our work, the GP application with probabilistic interpretation answers both verification and determination based on a combination of SIRA, TEA and genus distribution.

A natural first step in solving a timber harvest location question is to compare the data from the test sample to the reference data from the claimed origin (‘verification’). Having samples from every location within a species range is difficult, and in some cases only reference data from the claimed origin can be used to scrutinize a claim. However, confidently rejecting, for example, a country origin claim requires that every single possible harvest location within that country can be rejected. We evaluated tests based on SIRA, TEA and both data types combined. Our approach considers every possible harvest location within a country. The results (Fig. 2) illustrate the potential of statistical verification methods for tracing conflict timber, while underscoring the limits of SIRA/TEA-based verification as a tool to distinguish between relatively close locations. The test becomes more powerful when considering smaller harvest location claims (sensitivity increased from 39.9% on the country level to 59.6% on the 0.25° × 0.25° concessions level; Supplementary Fig. 1), which is to be expected as less surface area needs to be rejected as potential origin. This proves especially powerful considering the newly adopted EUDR, which requires operators to report plot Global Positioning System data or polygon data when introducing timber to the European Union market.

A more naive approach to test the hypotheses in equation (1) is to model samples within a country using a single probability distribution (for example, multivariate normal) and disregard the spatial heterogeneity of samples. While conceptually simpler, such tests are likely to wrongly reject a test sample originating from a poorly sampled region or country *c*, especially for large countries with diverse climatic conditions. By contrast, our GP model adjusts for the higher uncertainty in poorly sampled locations, which should lead to a lower type-I error rate.

One limitation of verification methods is that they model the distribution of SIR and TE values inside the claimed location of origin, while essentially disregarding information from other locations. Determination methods can leverage larger data sets from wider ranges to infer the likely harvest location. Elemental tracing outperforms SIRA when looking at finer spatial scales^15^, and previous work using TEA to trace timber sample origin could differentiate concessions 50 km apart^15^. Our best TEA model has an average error of 199.82 km. It is crucial to highlight that, unlike in the study by Boeschoten et al.^15^, our research treats the determination problem as a regression problem within a continuous space compared to a classification problem with fixed options.

To identify resolution limits of a continuous space modelling approach, an understanding of the underlying environmental and climatic effects is crucial. Hydrogen and oxygen stable isotopes in plants originate from soil-derived water, and thus from precipitation^23^, with two key effects being the latitude and continental effect (Siegwolf et al.^11^ and Gat^24^). Within the determination model, both *δ*^2^H and *δ*^18^O have a large impact on latitude. Plant carbon isotope variability is related to atmospheric carbon isotope composition, and intercellular CO_2_ concentration during the growing season strongly affects plant *δ*^13^C variability^11,25^. Within our findings, *δ*^13^C has a large impact on latitude for *Fagus* and on longitude for *Quercus*. The common elements between our work, Boeschoten et al.^17^ and Rees^16^ are Ca, Sr and Zn. A critical distinction is that our study area encompassed Eastern Europe, while Boeschoten et al.^17^ and Rees^16^ focused on Africa. In European topsoils, the elemental distribution follows geochemical variation patterns explained by processes occurring at different spatial scales (Imrie et al.^26^) (for a comparison of total versus location variation of key variables, see Section 3 in Supplementary Information). Large autocorrelation ranges were found for Ni due to mineralization and for Pb due to anthropogenic pollution, whereas smaller ranges were obtained for Cu and Zn^27^. Within our models, Ni appears to be important towards latitude for *Betula* and latitude and longitude for *Fagus*, whereas Zn is important towards longitude for *Pinus*. The bioavailability of Ni varies with pH, organic matter content, clay and iron oxides/hydroxides^28,29^. Future research should focus on incorporating potential trace element variability, especially in relation to the mobilization of trace elements within trees. Radial patterns of Cd, Pb and Zn concentrations were detected in *Fagus sylvatica* stem wood^30^; these variations should be incorporated in future models. In our method, trace element data are measured on multiple growth rings milled together, and as such obtains average values and discards any potential temporal or radial variability.

Our pathway allows accurate timber harvest location verification and determination and provides a tool to scrutinize harvest location claims. The verification method rejects 40% of false country-level claims while maintaining a low probability of rejecting correct claims. The accuracy of detecting false claims improves to 60% when operating at smaller spatial scales, showing the potential of scrutinizing plot claims under the EUDR. For samples from Russia and Belarus, our verification method detects, respectively, 82% and 47% of false claims, which underscores its potential for enforcing the current sanctions regime. These numbers are likely to further improve with increased sampling. Our determination method predicts harvest locations within 180 to 230 km of the true location, and the 95% confidence regions overlap country borders in some cases. More reference samples from sanctioned areas, including central and Eastern Russia, should be collected. By calculating SHAP values, we identify SIR and/or TE values with the most influence on latitude and longitude prediction. This aids in feature selection, model simplification and gaining deeper insights to the underlying relationships between stable isotopes and trace elements in timber harvest location. Future work should focus on incorporating potential trace element variability within wood and filling in data gaps by sampling across species ranges.

## Methods

### Sample collection and measurements

A total of 7,903 pith-to-bark samples were collected by Preferred by Nature from 3,867 trees, from 24 temperate species from 11 countries (Belarus, Croatia, Estonia, Finland, Hungary, Latvia, Lithuania, Moldova, Romania, Slovakia and Ukraine). We consider a larger study area and sampling strategy, allowing to capture more climatic variability. For each species, within each country, three trees were sampled within an area of 50 km; the next set of three trees was then 100 to 250 km away, keeping in mind the species distribution. A subset was chosen based on priority genera for timber traceability in Eastern Europe (*Betula*, *Fagus*, *Pinus* and *Quercus*), and 905 samples were shipped to Agroisolab GmbH in Julich, Germany, for SIRA and TEA (X-ray fluorescence). The subset contained *Betula pendula*, *Betula pubescens*, *Betula* sp., *F. sylvatica*, *Pinus nigra*, *Pinus* sp., *Pinus sylvestris*, *Quercus petraea*, *Quercus pubescens* and *Quercus robur*.

SIRA for carbon *δ*^13^C (ratio between ^13^C and ^12^C), hydrogen *δ*^2^H (ratio between ^2^H and ^1^H), nitrogen *δ*^15^N (ratio between ^15^N and ^14^N), oxygen *δ*^18^O (ratio between ^18^O and ^16^O) and sulfur *δ*^34^S (ratio between ^34^S and ^32^S) was undertaken following the methodology first developed to check the origin of timber from concessions in Cameroon^31^ and finalized in the International Tropical Timber Organization project in 2012 to develop and implement a timber tracking system with stable isotopes in Africa (ITTO PD 620/11 M). Afterwards, the methods were used for verifying the origin of oak from the USA^32^. The method includes the measurement of the isotope ratios of covalently bonded hydrogen after nitration as well (*δ*^2^H_nit_). In addition, ball-milled, oven-dried wood powder samples were subject to X-ray fluorescence spectroscopy (XEPOS, SPECTRO analytical instruments) analysis to determine the relative abundance of 17 trace elements (Al, Si, P, S, Cl, K, Ca, Mn, Fe, Ni, Cu, Zn, Br, Rb, Sr, Ba and Pb). The instrument is composed of an X-ray tube with a thick binary Pd/Co alloy anode in combination with adaptive excitation, resulting in low background and low detection limits. For sample comparisons, normalized net intensities were used, obtained by deconvolution from the measured spectra. Normalized net intensities remove influences originating from potentially different calibrations. Intensities were normalized on a Compton-scatter region in the spectra as internal standard, which results in relative TE concentrations. This approach corrects for matrix effects as described, for example, by Anderman and Kemp^33^. Two scatter regions with different energies were used as a correction. Scatter region 1 (6.75–6.83 keV) was for the elements Al, Si, P, S, Cl, K and Ca, and scatter region 2 (19.71–20.55 keV) was for the elements Mn, Fe, Ni, Cu, Zn, Br, Rb, Sr, Ba and Pb. As such, each tree sample therefore generated 23 chemical values to be analysed for comparative values at a given Global Positioning System point, representing a substantial improvement in sophistication and robustness in timber-tracking data sets.

This dataset was supplemented with 24 timber reference samples from Russia which were collected in previous projects and obtained through Agroisolab GmbH. The same SIRA and X-ray fluorescence spectroscopy analysis was performed on these samples.

### Data pre-processing

In the following sections, we used the notation *y*_*j* = 1,…,*m*_ for the *m* = 20 SIR/TE values. For the subsequent data analysis, Mn, Br and Ba were omitted due to a large proportion of missing values that were introduced by the process outlined in the previous section. Furthermore, we found that TE values varied across several orders of magnitude between samples, so we applied a log transformation on the trace element concentrations to stabilize the variance in the data and mitigate the impact of potential outliers.

Based on the spatial range of our collected samples, we defined a study area, denoted by ${{{\mathcal{X}}}}$, consisting of coordinates *x* = (*x*_lon_, *x*_lat_) (expressed in longitude and latitude). It is defined as a rectangular grid consisting of equally spaced locations between 15° and 39.5° longitude and 46° and 68.3° latitude while taking into account the genus range of interest (that is, coordinates that are not part of a genus range are excluded). Genus ranges were extracted from publicly available data in Caudullo et al.^34^. We chose a resolution of 0.2° (~25 km), which allowed us to approximate spatial probability distributions with high accuracy.

### Modelling of stable isotope ratios and trace elements

To tackle verification and determination, we used GP regression to model the spatial probability distribution of SIR and TE values inside the study area of interest. GPs are a powerful and flexible class of probabilistic models used in machine learning and statistics, both for regression and classification tasks. In this work, we used GP regression to estimate values at unobserved locations based on observations at nearby locations. Under a GP regression model, values of a variable measured at different spatial locations are assumed to be jointly normally distributed, with a covariance function specified by the model. This bears resemblance to kriging, a geostatistical interpolation technique commonly used in geoscience for spatial analysis^35^.

We fitted separate models for each genus we analysed. For every SIR/TE, we trained a GP regression model to predict its expected value at unobserved spatial locations. In essence, every GP regression model consists of three key components: (1) the prior mean function, for which we assume a constant value; (2) the covariance function, for which we use the Matérn function^19^ with separate scaling parameters for latitude and longitude in the determination model; and (3) the noise parameter. The selection of mean and covariance functions embodies our prior knowledge and modelling assumptions regarding the regression problem. The covariance function defines the similarity between data points in the spatial domain and allows GPs to capture different types of complex patterns and relationships in the data.

After training a GP regression model, we can predict the mean and variance for the *j*th SIR/TE value at a particular location *x*, which we denote by ${\hat{\mu }}_{j}({{{{x}}}})$ and ${\hat{\sigma }}_{j}^{2}({{{{x}}}})$, respectively. The predicted variance can be interpreted as the total uncertainty at location *x* and consists of aleatoric uncertainty and epistemic uncertainty^36^. The former is linked to inherently random effects in the SIR and TE values. It stems from natural variability in the data and reflects factors that are beyond our ability to control. The latter arises due to gaps in our knowledge and understanding. In the context of our model, it reflects the uncertainty associated with our limited information about SIR and TE values at different locations and can be reduced by acquiring more data. For a more formal discussion about GP regression for spatial modelling, we refer the reader to Williams and Rasmussen^19^.

### Verification

Ideally, a verification test should have high power in terms of detecting fraudulent cases while at the same time be reliable, in the sense of having a low risk of false accusations. A principled approach, therefore, consists of designing a statistical hypothesis test that can be used to answer the following set of hypotheses for a new test sample $({y}_{1}^{* },\ldots ,{y}_{m}^{* })$ of a given genus with a territorial unit claim *c*:

1 $$\begin{array}{l}{H}_{0}:{{{\rm{The}}}}\,{{{\rm{test}}}}\,{{{\rm{sample}}}}\,({y}_{1}^{* },\ldots ,{y}_{m}^{* })\\\qquad{{{\rm{comes}}}}\,{{{\rm{from}}}}\,{{{\rm{territorial}}}}\,{{{\rm{unit}}}}\,c.\\ {H}_{1}:{{{\rm{The}}}}\,{{{\rm{test}}}}\,{{{\rm{sample}}}}\,({y}_{1}^{* },\ldots ,{y}_{m}^{* })\\\qquad{{{\rm{does}}}}\,{{{\rm{not}}}}\,{{{\rm{come}}}}\,{{{\rm{from}}}}\,{{{\rm{territorial}}}}\,{{{\rm{unit}}}}\,c\,.\end{array}$$

With a territorial unit considered as any possible spatial scale attached to a harvest location claim (for example, country, region, concession, plot…), the statistical hypothesis test should allow the user to control the type I error rate by means of the significance level *α*. It is the probability of rejecting the null hypothesis *H*_0_ when it is true (or the probability of making a false accusation). The significance level allows the user to define a trade-off between the two important aforementioned criteria, namely, reliability and power: the higher the significance level *α*, the lower the reliability, yet the higher the power of the test to detect fraudulent cases. In this work, we propose a statistical hypothesis test based on the Gaussian process regression model that was defined in the previous section. In particular, for a new test sample $({y}_{1}^{* },\ldots ,{y}_{m}^{* })$ of a given genus with territorial unit claim *c* and corresponding area ${{{{\mathcal{X}}}}}_{c}$ (subset of study area ${{{\mathcal{X}}}}$ consisting of locations in territorial unit *c*), we consider a composite hypothesis test of the form

$$\begin{array}{l}{H}_{0}:{\exists }_{{{{{x}}}}\in {{{{\mathcal{X}}}}}_{c}}{\forall }_{j\in \left\{1,\ldots ,m\right\}}{y}_{j}^{* } \sim {{{\mathcal{N}}}}({\hat{\mu }}_{j}({{{{x}}}}),{\hat{\sigma }}_{j}^{2}({{{{x}}}}))\\ {H}_{1}:\neg {H}_{0}\end{array}$$

with ${{{\mathcal{N}}}}({\hat{\mu }}_{j}({{{{x}}}}),{\hat{\sigma }}_{j}^{2}({{{{x}}}}))$ a Gaussian distribution with mean ${\hat{\mu }}_{j}({{{{x}}}})$ and ${\hat{\sigma }}_{j}^{2}({{{{x}}}})$. In other words, under the null hypothesis, we assume that the *j*th ratio value for the test sample can come from any location in the territorial unit area ${{{{\mathcal{X}}}}}_{c}$. To reject the declared origin, we must thus reject every location in ${{{{\mathcal{X}}}}}_{c}$. Furthermore, as we assume independence between different stable isotopes and trace elements, under *H*_0_ we have that

$$S({{{{x}}}})=\mathop{\sum }\limits_{j=1}^{m}\frac{{\left({y}_{j}^{* }-{\hat{\mu }}_{j}({{{{x}}}})\right)}^{2}}{{\hat{\sigma }}_{j}^{2}({{{{x}}}})}$$

is a sum of independent squared standard Gaussian distributions, which follows a chi-squared distribution with *m* degrees of freedom for some ${{{{x}}}}\in {{{{\mathcal{X}}}}}_{c}$. As rejecting the null hypothesis amounts to rejecting for every point in ${{{{\mathcal{X}}}}}_{c}$, we take a maximum of *P* values across the claimed territorial unit area (ref. ^37^, chap. 10):

$$p=\mathop{\max }\limits_{{{{{x}}}}\in {{{{\mathcal{X}}}}}_{c}}[1-F(S({{{{x}}}}))]\,,$$

where *F* is the cumulative distribution function of a chi-squared distribution with *m* degrees of freedom.

### Determination

In contrast to verification in the previous section, the goal in determination is to predict the timber harvest location of a new test sample from a particular genus. We propose a Bayesian determination model based on GP regression. For a test sample ${{{{{y}}}}}^{* }=({y}_{1}^{* },\ldots ,{y}_{m}^{* })$ and *m* trained GP regression models (‘Modelling of stable isotope ratios and trace elements’), the posterior probability of a location ${{{{x}}}}\in {{{\mathcal{X}}}}$ is calculated by means of applying Bayes’ rule:

2 $$Pr[{{{{x}}}}\,| \,{{{{{y}}}}}^{* }]=\frac{{{{\mathcal{L}}}}({{{{{y}}}}}^{* }\,| \,{{{{x}}}})Pr[{{{{x}}}}]}{{\sum }_{{{{{{x}}}}}^{{\prime} }\in {{{\mathcal{X}}}}}{{{\mathcal{L}}}}({{{{{y}}}}}^{* }\,| \,{{{{{x}}}}}^{{\prime} })Pr[{{{{{x}}}}}^{{\prime} }]}\,,$$

where the likelihood function is given by:

$${{{\mathcal{L}}}}({{{{y}}}}\,| \,{{{{x}}}})=\mathop{\prod }\limits_{j=1}^{m}\phi ({y}_{j}\,| \,{\hat{\mu }}_{j}({{{{x}}}}),{\hat{\sigma }}_{j}^{2}({{{{x}}}}))\,,$$

with *ϕ*(. ∣ *a*, *b*) the probability density function of a Gaussian distribution with mean *a* and variance *b*. For the prior distribution *Pr*[*x*], we consider a uniform prior for locations ${{{{x}}}}\in {{{\mathcal{X}}}}$ that are within a range of 300 km to the reference data, and a zero prior elsewhere. The rationale behind this prior is to restrict determination to regions containing reference data. The posterior probability in equation (2) denotes the (posterior) probability that the test sample y* originates from location ${{{{x}}}}\in {{{\mathcal{X}}}}$. After calculating the posterior probability for every location in the study area ${{{\mathcal{X}}}}$, we report the predicted timber harvest location and the 95% confidence region for every test sample y*. The former corresponds to the location with highest posterior probability. The latter corresponds to the smallest set of locations whose posterior probability is at least 95% according to the model. Confidence regions are equivalent to credible regions in Bayesian statistics and correspond to regions within which the true location falls with 95% probability^38^. The interpretation is that there is a 95% probability that the true timber harvest location lies within the 95% confidence region, given the evidence provided by the observed data. Note that the confidence region is constructed based on the posterior distribution in equation (2) and therefore depends on the prior *Pr*[*x*], the likelihood ${{{\mathcal{L}}}}(\;{{{{{y}}}}}^{* }\,| \,{{{{x}}}})$ of the observed data and model correctness. To gain further insights in the 95% confidence regions, we have conducted additional experiments showcasing the efficiency and coverage of the predicted 95% confidence regions, obtained on the test samples (Section 2 in Supplementary Information).

To quantify the relative influence of different SIR and TE on the accuracy of timber harvest location determination, we calculated SHAP values for the mode of the posterior distribution in equation (2) (ref. ^39^). Given SIR and TE values and a determination model (that is, mode of the posterior distribution), SHAP approximates the determination model. By means of the approximation, a SHAP value, which represents the marginal contribution of a given stable isotope or trace element value to the output of the determination model, is calculated. The SHAP value is analogous to the Shapley value in cooperative game theory and offers a principled approach for interpreting the contribution of specific stable isotopes and trace elements for timber harvest location determination.

### Experimental set-up

We trained and validated three different determination models for every genus by means of fourfold cross-validation on the reference samples. In particular, we considered determination models based on (1) SIR data only, (2) TE concentration data only and (3) a combination of both data types. For every training iteration, we standardized the SIR and/or TE values and estimated the parameters of the GP regression models, along with the noise parameter, by maximizing the likelihood on the training fold. This approach stands in contrast to traditional kriging approaches in the geostatistics literature, which rely on approximate techniques based on summary statistics. We used Adam optimization with a learning rate of 0.01 and the Stochastic Gradient Descent with Warm Restarts scheduler and early stopping with a patience of five iterations^40,41^. In terms of performance for all determination models, we report the average and standard deviation of the great-circle distance between true and predicted locations, averaged over all test folds.

In another set of experiments, we highlighted how insights can be derived from SHAP values regarding the impact of SIR and TE values on determination. To this extent, we used 80% of the reference samples for training GP regression models for all SIR and TE values, as outlined above. The remaining samples were then used as test samples for which we constructed determination maps by calculating the posterior probability in equation (2) for every point in the study area. For the sake of interpretation, we report 95% confidence regions, that is, sets of locations whose total posterior probability exceeds 95%. In addition, we also present uncertainty maps for all genera by calculating, for every location in the study area, the logarithm of the product of predicted variances for all SIR and TE values (‘Modelling of stable isotope ratios and trace elements’). When it comes to the SHAP analysis, we calculated SHAP values on the test set and present beeswarm plots for every genus^39^.

We used the same cross-validation and grid set-up to evaluate the performance of our verification procedure. Due to space constraints, we focus on *Betula* samples. For every test sample *y* = (*y*_1_, …, *y*_*m*_) and every country *c* in the study, we tested the hypothesis that y originates from *c*. The set of within-country locations ${{{{\mathcal{X}}}}}_{c}$ is defined as the set of all grid points within country *c* for all countries except Russia and Ukraine, where we only have samples from several regions. For those countries, we defined the set of allowed within-country locations as the set of grid points within the level-1 administrative subdivisions from which we have samples. For each country, we report the following: (1) specificity (the probability of not rejecting a correct origin claim) and (2) sensitivity (the probability of rejecting an incorrect origin claim). We also report the overall accuracy for each combination of true and claimed origin. In all cases, a claim is rejected whenever the *P* value is lower than *α* = 0.05.

To investigate the impact of the scale of claimed locations on verification accuracy, we repeated the verification analysis at different spatial scales (Section 1 in Supplementary Information). For each simulated incorrect country claim, we randomly sampled a location within that country and identified the territorial unit corresponding to the location. For correct country claims, territorial units corresponding to the true location were used. We then performed verification tests at the level of territorial units. Three spatial scales were used: level-1 administrative units and simulated forest concessions of size 0.5° × 0.5° and 0.25° × 0.25°, respectively. We reported sensitivity and specificity as in the country-level analysis. All experiments were performed in Python (version 3.10.9) using the GPyTorch (version 1.10), PyTorch (version 1.13.1), Scikit-learn (version 1.2.1), SHAP (version 0.41.0), Skorch (version 0.12.1) and matplotlib (version 3.5.0) libraries^39,42–46^.

### Availability of materials

The wood samples are part of the World Forest ID Georeferenced Sample Collection. For access enquiries, please contact the corresponding author.

### Reporting summary

Further information on research design is available in the Nature Portfolio Reporting Summary linked to this article.

### Supplementary information

Supplementary Information Supplementary text, Supplementary Tables 1 and 2 and Supplementary Figs. 1–5.

Reporting Summary

## Data Availability

Due to the sensitive nature of the data, the SIRA and TEA data are available upon reasonable request.

## Code availability

The code is available upon request at https://zenodo.org/records/10055203.
