# Supplementary material for: A framework for tracing timber following the Ukraine invasion
Source: Nat Plants. 2024 Mar 11;10(3):390–401. doi: 10.1038/s41477-024-01648-5 (PMC10954544; doi:10.1038/s41477-024-01648-5)
Supplement: Supplementary file 1 — Supplementary text, Supplementary Tables 1 and 2 and Supplementary Figs. 1–5. [file 41477_2024_1648_MOESM1_ESM.pdf]

nature plants

Article

https://doi.org/10.1038/s41477-024-01648-5

# A framework for tracing timber following the Ukraine invasion

In the format provided by the authors and unedited

# Contents

**Section 1 Spatial scale analysis for verification** **2**

**Section 2 Efficiency and coverage of 95% confidence region in determination** **2**

**Section 3 Descriptive analysis of stable isotope ratio and element variation** **4**

# Section 1 Spatial scale analysis for verification

We generated simulated harvest location claims in a way that facilitates direct comparison of results between spatial scales. For each incorrect claim of country origin, a location was chosen uniformly at random from within the country. We then identified the level-1 administrative region containing the location and used that as a region-level harvest location claim. We simulated concession-level harvest location claims by choosing, uniformly at random, a rectangular area of specified size that contained the sampled location. Two concession sizes were investigated: 0.5 x 0.5 degrees and 0.25 x 0.25 degrees. For correct country origin claims, we chose region- and concession-level harvest location claims by applying the same procedure to the true location. In a second experiment, we limited location sampling for incorrect claims to those level-1 administrative regions that contained at least one training data point. This was done to investigate the impact of data availability near claimed locations on the sensitivity of the test.

The results are shown in Figure S1. The average sensitivity increases from 40% to 52% when level-1 units are used as declared harvest locations, rather than whole countries. For 0.25x0.25 degree concession harvest claims, the sensitivity increases to 60%. For samples from Russia, sensitivity was considerably higher at every spatial scale, ranging from 59% at country level to 82% at the concession level. This is accompanied by a slight decrease of specificity from 96% to 90% (see Figure S1). For samples from Belarus, the sensitivity ranged from 31% at country level to 51% at 0.5x0.5 concession level, with specificities between 100% and 85%. The incorrect harvest locations claims were more likely to be rejected if they were made in areas where training samples were available to our model - when incorrect claims were simulated only within level-1 units where at least one training sample has been collected, the sensitivity rose to 58% for level-1 declared locations, and 67% for 0.25x0.25 degree concessions across all countries and reached 87% and 62% for samples from Russia and Belarus, respectively. This suggests that the accuracy of our verification test might be improved by collecting additional samples in areas where false harvest location claims are frequently made.

# Section 2 Efficiency and coverage of 95% confidence region in determination

We recall the definition of the posterior probability of a location $\boldsymbol{x} \in \mathcal{X}$:

$$Pr[\boldsymbol{x} \mid \boldsymbol{y}^*] = \frac{\mathcal{L}(\boldsymbol{y}^* \mid \boldsymbol{x}) Pr[\boldsymbol{x}]}{\sum_{\boldsymbol{x}' \in \mathcal{X}} \mathcal{L}(\boldsymbol{y}^* \mid \boldsymbol{x}') Pr[\boldsymbol{x}']} , \tag{1}$$

where the likelihood function is given by:

$$\mathcal{L}(\boldsymbol{y} \mid \boldsymbol{x}) = \prod_{j=1}^{m} \phi(y_j \mid \hat{\mu}_j(\boldsymbol{x}), \hat{\sigma}_j^2(\boldsymbol{x})) ,$$

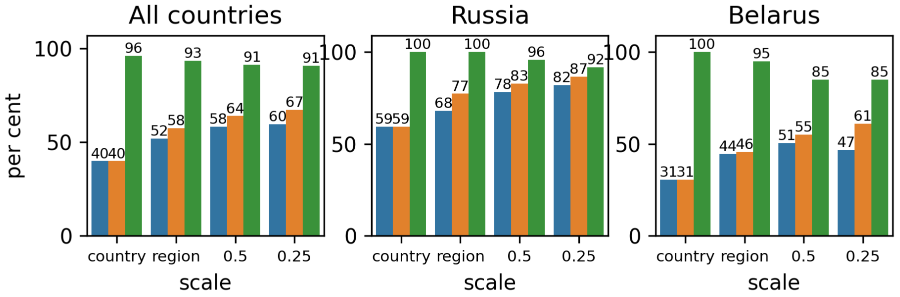


**Figure S1: Accuracy at different spatial scales for the whole dataset, as well as for samples from Belarus and Russia.** We report sensitivity for claims across each country (blue), sensitivity for claims within regions where samples are available (orange) and specificity (green) for four types of location claims: countrywide, regional (level-1 units), and simulated forest concessions of size 0.5x0.5 degrees and 0.25x0.25 degrees. More precise location claims result in improved sensitivity.

with $\phi(.\,|\,a, b)$ the probability density function of a Gaussian distribution with mean $a$ and variance $b$.

After calculating the posterior probability for every location in the study area $\mathcal{X}$, we report the predicted timber harvest location and the 95% confidence region for every test sample $\boldsymbol{y}^*$. The former corresponds to the location with highest posterior probability. The latter corresponds to the smallest set of locations whose posterior probability is at least 95% according to the model. Confidence regions are equivalent to credible regions in Bayesian statistics and correspond to regions within which the true location falls with 95% probability (**lee1989bayesian**). The interpretation is that there is a 95% probability that the true timber harvest location lies within the 95% confidence region, given the evidence provided by the observed data. Note that the confidence region is constructed based on the posterior distribution in Eqn. 1 and therefore depends on the prior $Pr[\boldsymbol{x}]$, the likelihood $\mathcal{L}(\boldsymbol{y}^*\,|\,\boldsymbol{x})$ of the observed data and model correctness.

To gain further insights in the 95% confidence regions, we have conducted additional experiments showcasing the *efficiency* and *coverage* of the predicted 95% confidence regions, obtained on the test samples. The former metric is expressed in km$^2$ and represents the average area of the 95% confidence regions. The latter metric reflects the proportion of test samples that encompass the true location. The results are presented in Table S1. In summary, the efficiency is given by 333006 km$^2$ (approx. 577 km $\times$ 577 km), 230274 km$^2$ (approx. 480 km $\times$ 480 km), 429200 km$^2$ (approx. 655 km $\times$ 655 km) and 274577 km$^2$ (approx. 524 km $\times$ 524 km), for *Betula*, *Fagus*, *Quercus* and *Pinus*, respectively. Note that the the total area of the study area, considered in this work, is approximately 5135505 km$^2$. The coverage is given by 92%, 90.91%, 91.38% and 84.09%, for *Betula*, *Fagus*, *Quercus* and *Pinus*, respectively. In most cases, the coverage is close to the target confidence level of 95%. However, it's crucial to emphasize that in real-world scenarios, the coverage

may deviate from the user-defined confidence level, due to the reasons that were outlined above: choice of prior, the observed data and model correctness.

**Table S1:** Efficiency and coverage for the 95% confidence regions obtained by different determination models on test data. We used the same test data, as outlined in the Experimental Setup in the main manuscript.

| Genus | Efficiency ($km^2$) | Coverage |
|---|---|---|
| *Betula* | 333006 | 92.00% |
| *Fagus* | 230274 | 90.91% |
| *Quercus* | 429200 | 91.38% |
| *Pinus* | 274577 | 84.09% |

# Section 3 Descriptive analysis of stable isotope ratio and element variation

In an additional set of experiments, we investigate the total and local variation of stable isotope ratios and trace elements, given the observed data for *Betula*, *Fagus*, *Pinus* and *Quercus*. The results are presented in Fig. S2, S3, S4 and S5, respectively. For the sake of space, based on insights from **boeschoten2022clay**, **Rees2015** and the feature importance insights from the SHAP analysis, the following subset of the stable isotope ratios and trace elements are chosen: $\delta^2$H, $\delta^2$H$_{nit}$, $\delta^{13}$C, $\delta^{15}$N, $\delta^{18}$O, $\delta^{34}$S, Ca, Cl, Cu, Fe, Ni, Pb, Sr and Zn.

We generate boxplots to assess the total versus local variability of the subset of stable isotope ratios and trace elements for each genus. In each plot, the left panel presents the total variation, i.e., encompassing all available data, while the right panel presents local variations specific to each country. To analyze local variations, we use 4-means clustering based on the Euclidean distance, categorizing test samples into four distinct clusters. Each cluster contains samples that are closely located in space. Expanding the number of clusters enhances our understanding of the local variation in specific stable isotopes or trace elements within a particular country.

The following main trends are observed: 1) local variations tend to be notably smaller than total variations, and 2) in most cases, a substantial difference in average stable isotope and trace element values exists between different countries and clusters within countries. This illustrates that the majority of stable isotope ratios and trace elements contain sufficient spatial information for most genera.

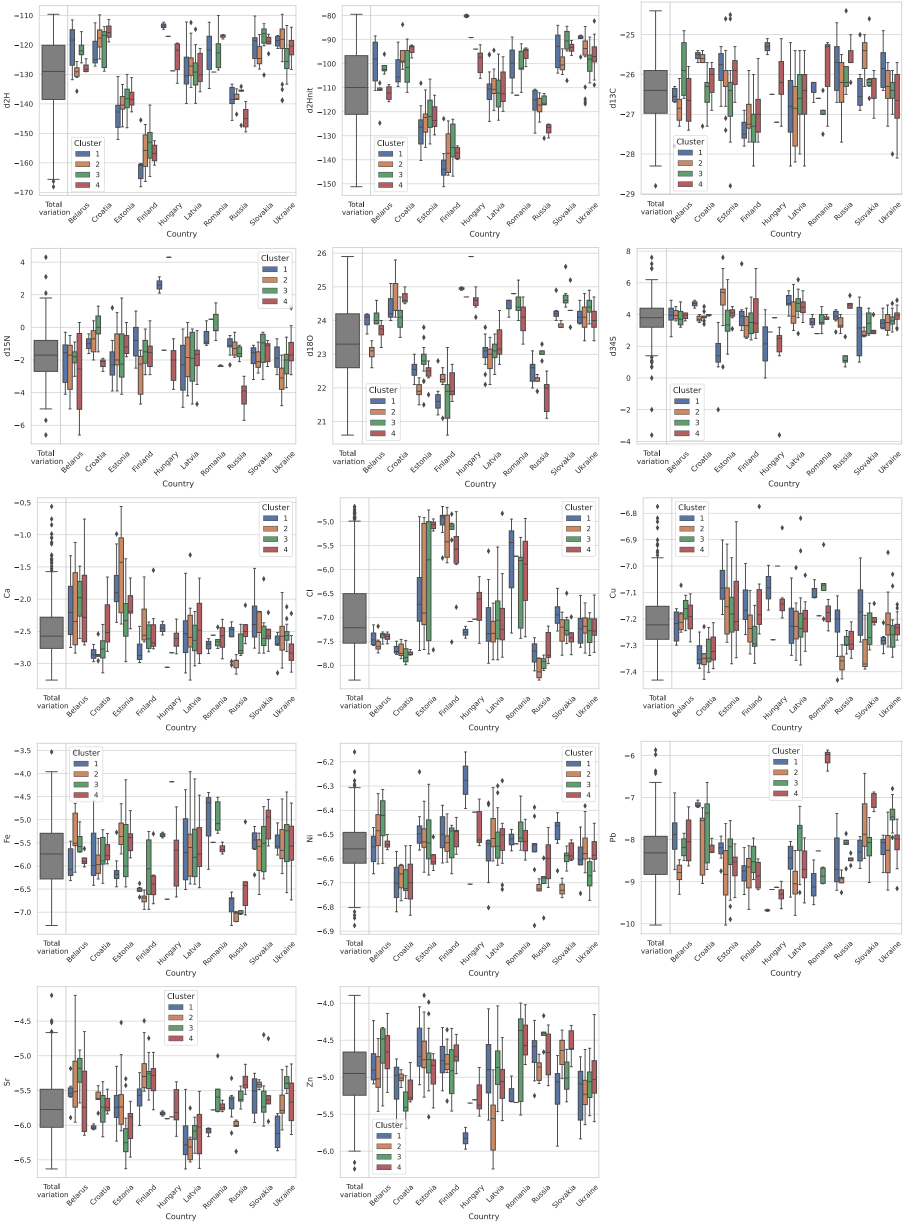


**Figure S2: Total and local variation for different stable isotopes and trace elements in Betula.** Stable isotopes ($\delta^2$H, $\delta^2$H$_{nit}$, $\delta^{13}$C, $\delta^{15}$N, $\delta^{18}$O and $\delta^{34}$S) and trace elements (Ca, Cl, Cu, Fe, Ni, Pb, Sr and Zn) are considered. Local variation is plotted by means of boxplots for four distinct clusters, obtained by 4-means clustering and the Euclidean distance, in each country. Total variation is plotted by means of a boxplot for all data. The boxplots show the median and 25th and 75th percentiles of the data, with whiskers extending to 1.5 times the interquartile range. Sample sizes for the boxplots are presented in Table S2.

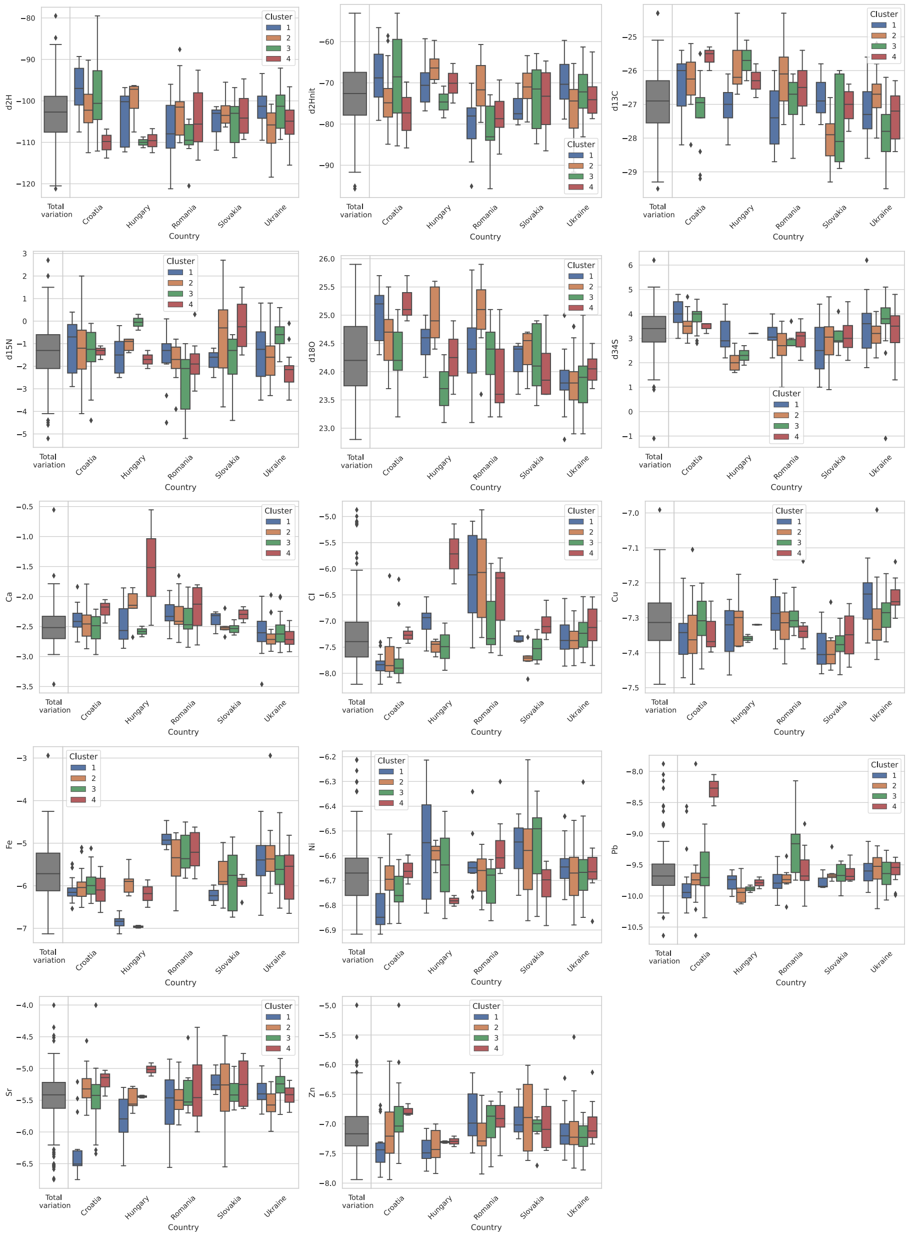

**Figure S3: Total and local variation for different stable isotopes and trace elements in Fagus.** Stable isotopes ($\delta^2$H, $\delta^2$H$_{nit}$, $\delta^{13}$C, $\delta^{15}$N, $\delta^{18}$O and $\delta^{34}$S) and trace elements (Ca, Cl, Cu, Fe, Ni, Pb, Sr and Zn) are considered. Local variation is plotted by means of boxplots for four distinct clusters, obtained by 4-means clustering and the Euclidean distance, in each country. Total variation is plotted by means of a boxplot for all data. The boxplots show the median and 25th and 75th percentiles of the data, with whiskers extending to 1.5 times the interquartile range. Sample sizes for the boxplots are presented in Table S2.

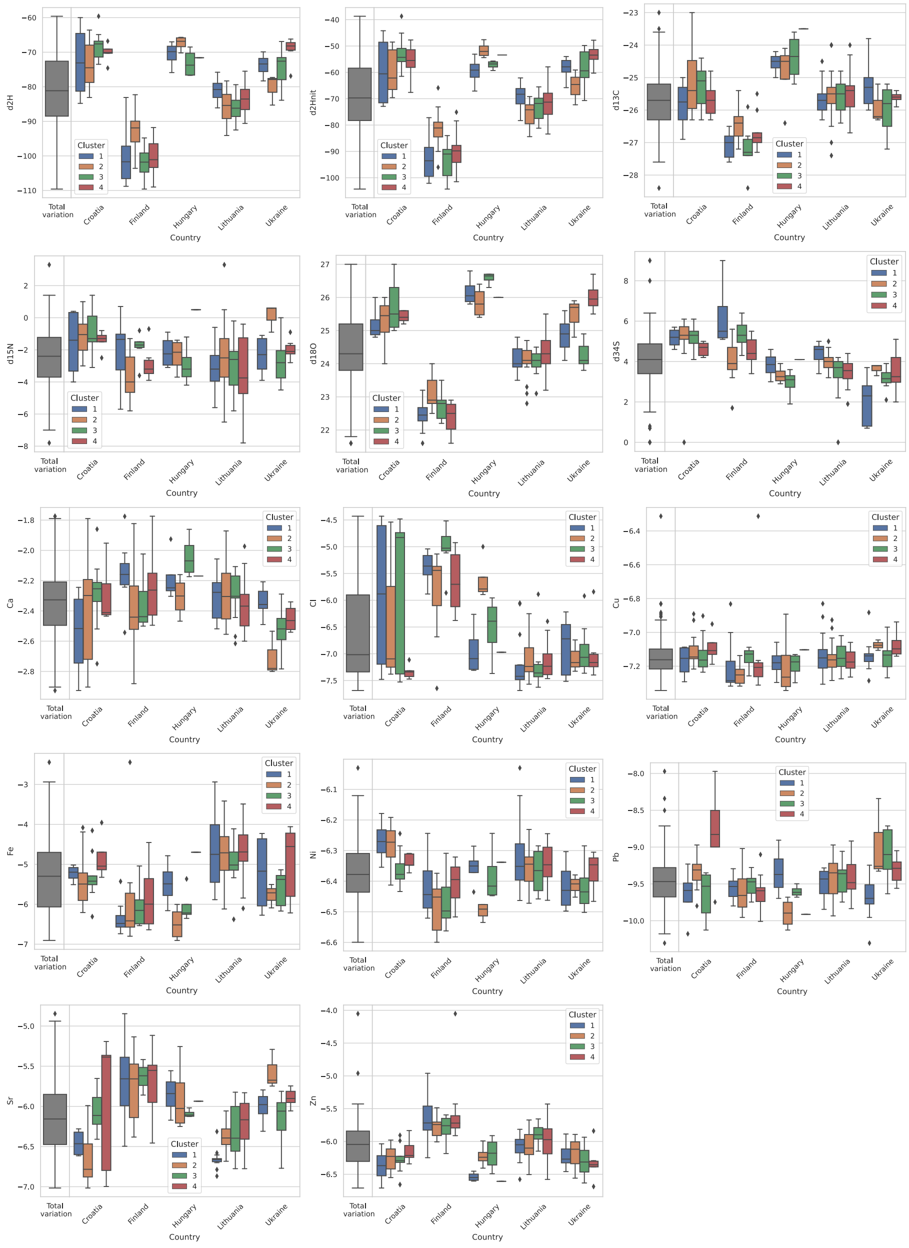

**Figure S4: Total and local variation for different stable isotopes and trace elements in Pinus.** Stable isotopes ($\delta^2$H, $\delta^2$H$_{nit}$, $\delta^{13}$C, $\delta^{15}$N, $\delta^{18}$O and $\delta^{34}$S) and trace elements (Ca, Cl, Cu, Fe, Ni, Pb, Sr and Zn) are considered. Local variation is plotted by means of boxplots for four distinct clusters, obtained by 4-means clustering and the Euclidean distance, in each country. Total variation is plotted by means of a boxplot for all data. The boxplots show the median and 25th and 75th percentiles of the data, with whiskers extending to 1.5 times the interquartile range. Sample sizes for the boxplots are presented in Table S2.

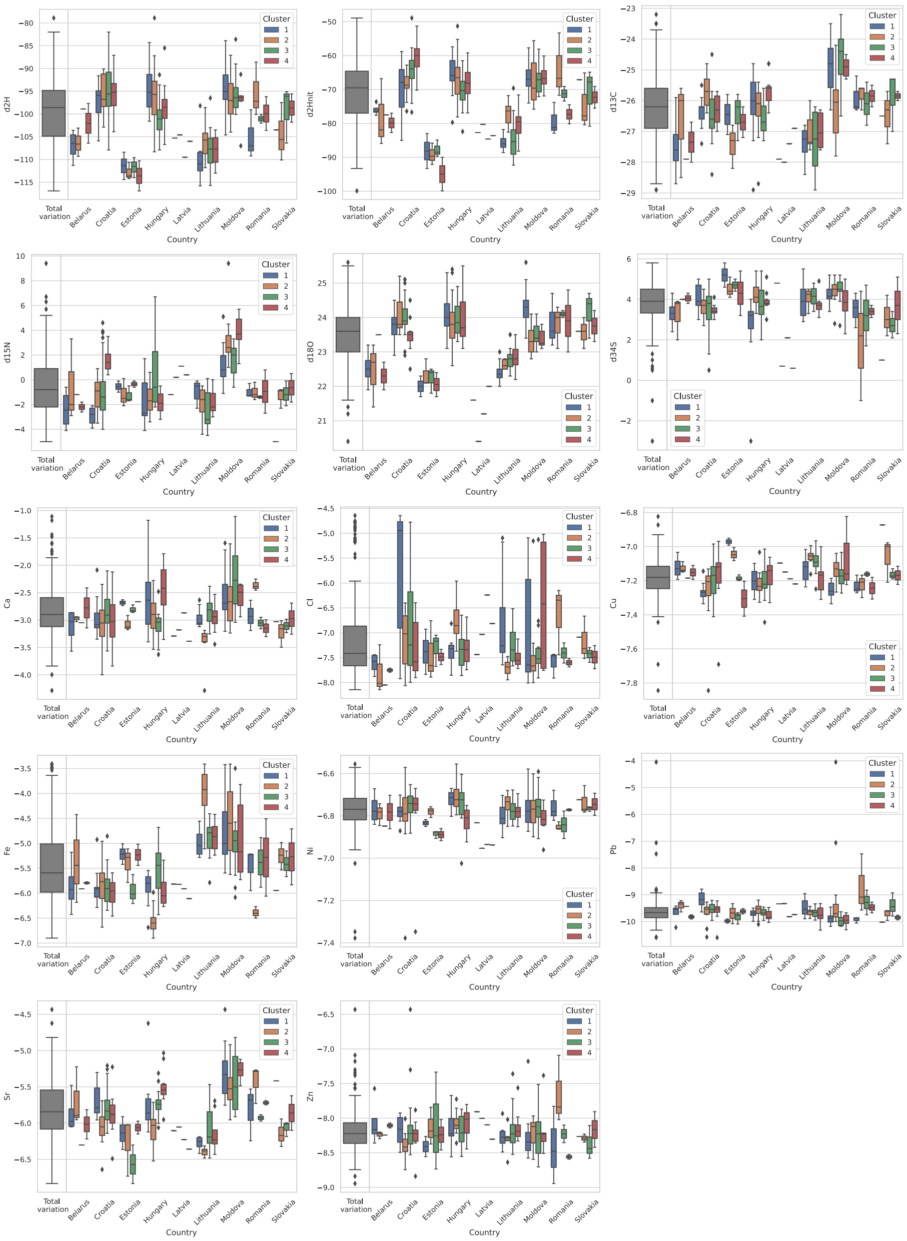


**Figure S5: Total and local variation for different stable isotopes and trace elements in Quercus.** Stable isotopes ($\delta^2$H, $\delta^2$H$_{nit}$, $\delta^{13}$C, $\delta^{15}$N, $\delta^{18}$O and $\delta^{34}$S) and trace elements (Ca, Cl, Cu, Fe, Ni, Pb, Sr and Zn) are considered. Local variation is plotted by means of boxplots for four distinct clusters, obtained by 4-means clustering and the Euclidean distance, in each country. Total variation is plotted by means of a boxplot for all data. The boxplots show the median and 25th and 75th percentiles of the data, with whiskers extending to 1.5 times the interquartile range. Sample sizes for the boxplots are presented in Table S2.

**Table S2:** Sample sizes for descriptive analysis of stable isotope ratio and element variation. For each country and genus, we show the sample sizes for the four clusters.

| Genus | *Betula* | | | | *Fagus* | | | | *Pinus* | | | | *Quercus* | | | |
|---|---|---|---|---|---|---|---|---|---|---|---|---|---|---|---|---|
| Belarus | 6 | 6 | 4 | 4 | 0 | 0 | 0 | 0 | 0 | 0 | 0 | 0 | 4 | 3 | 1 | 2 |
| Croatia | 3 | 5 | 9 | 3 | 15 | 24 | 18 | 3 | 4 | 12 | 9 | 5 | 9 | 11 | 27 | 10 |
| Estonia | 12 | 13 | 18 | 7 | 0 | 0 | 0 | 0 | 0 | 0 | 0 | 0 | 2 | 3 | 3 | 2 |
| Finland | 7 | 8 | 9 | 6 | 0 | 0 | 0 | 0 | 8 | 11 | 7 | 10 | 0 | 0 | 0 | 0 |
| Hungary | 2 | 1 | 1 | 6 | 7 | 5 | 2 | 2 | 4 | 4 | 4 | 1 | 11 | 15 | 12 | 11 |
| Latvia | 15 | 12 | 17 | 12 | 0 | 0 | 0 | 0 | 0 | 0 | 0 | 0 | 1 | 1 | 1 | 1 |
| Lithuania | 0 | 0 | 0 | 0 | 0 | 0 | 0 | 0 | 15 | 21 | 13 | 16 | 8 | 6 | 12 | 8 |
| Moldova | 0 | 0 | 0 | 0 | 0 | 0 | 0 | 0 | 0 | 0 | 0 | 0 | 18 | 12 | 15 | 6 |
| Romania | 3 | 1 | 5 | 3 | 10 | 11 | 7 | 7 | 0 | 0 | 0 | 0 | 3 | 3 | 2 | 2 |
| Russia | 9 | 5 | 5 | 5 | 0 | 0 | 0 | 0 | 0 | 0 | 0 | 0 | 0 | 0 | 0 | 0 |
| Slovakia | 6 | 3 | 6 | 5 | 3 | 6 | 7 | 4 | 0 | 0 | 0 | 0 | 1 | 3 | 3 | 2 |
| Ukraine | 6 | 21 | 15 | 18 | 28 | 21 | 27 | 12 | 9 | 3 | 12 | 6 | 0 | 0 | 0 | 0 |
