# Supplementary material for: A framework for tracing timber following the Ukraine invasion
Source: Nat Plants. 2024 Mar 11;10(3):390–401. doi: 10.1038/s41477-024-01648-5 (PMC10954544; doi:10.1038/s41477-024-01648-5)
Supplement: Supplementary file 2 — Reporting Summary [file 41477_2024_1648_MOESM2_ESM.pdf]

nature portfolio

Corresponding author(s): Victor Deklerck

Last updated by author(s): Jan 29, 2024

Reporting Summary

Nature Portfolio wishes to improve the reproducibility of the work that we publish. This form provides structure for consistency and transparency in reporting. For further information on Nature Portfolio policies, see our Editorial Policies and the Editorial Policy Checklist.

Statistics

For all statistical analyses, confirm that the following items are present in the figure legend, table legend, main text, or Methods section.

n/a	Confirmed	
☐	☒	The exact sample size (*n*) for each experimental group/condition, given as a discrete number and unit of measurement
☐	☒	A statement on whether measurements were taken from distinct samples or whether the same sample was measured repeatedly
☐	☒	The statistical test(s) used AND whether they are one- or two-sided<br>*Only common tests should be described solely by name; describe more complex techniques in the Methods section.*
☒	☐	A description of all covariates tested
☐	☒	A description of any assumptions or corrections, such as tests of normality and adjustment for multiple comparisons
☒	☐	A full description of the statistical parameters including central tendency (e.g. means) or other basic estimates (e.g. regression coefficient) AND variation (e.g. standard deviation) or associated estimates of uncertainty (e.g. confidence intervals)
☐	☒	For null hypothesis testing, the test statistic (e.g. F, t, r) with confidence intervals, effect sizes, degrees of freedom and P value noted<br>*Give P values as exact values whenever suitable.*
☐	☒	For Bayesian analysis, information on the choice of priors and Markov chain Monte Carlo settings
☒	☐	For hierarchical and complex designs, identification of the appropriate level for tests and full reporting of outcomes
☒	☐	Estimates of effect sizes (e.g. Cohen's *d*, Pearson's *r*), indicating how they were calculated

Our web collection on statistics for biologists contains articles on many of the points above.

Software and code

Policy information about availability of computer code

Data collection	No software was used for data collection.
Data analysis	The code is available upon request at https//zenodo.org/records/10055203. The code was developed in Python (version 3.10.9) based on the following libraries: Pytorch (version 3.10.9), GPytorch (version 1.10), Scikit-learn (version 1.2.1), Skorch (0.12.1), matplotlib (version 3.5.0) and SHAP (version 0.41.0).

For manuscripts utilizing custom algorithms or software that are central to the research but not yet described in published literature, software must be made available to editors and reviewers. We strongly encourage code deposition in a community repository (e.g. GitHub). See the Nature Portfolio guidelines for submitting code & software for further information.

Data

Policy information about availability of data

All manuscripts must include a data availability statement. This statement should provide the following information, where applicable:

- Accession codes, unique identifiers, or web links for publicly available datasets
- A description of any restrictions on data availability
- For clinical datasets or third party data, please ensure that the statement adheres to our policy

Due to the sensitive nature of the data, the SIRA and TEA data are available upon request.

Research involving human participants, their data, or biological material

Policy information about studies with human participants or human data. See also policy information about sex, gender (identity/presentation), and sexual orientation and race, ethnicity and racism.

Reporting on sex and gender	N/A
Reporting on race, ethnicity, or other socially relevant groupings	N/A
Population characteristics	N/A
Recruitment	N/A
Ethics oversight	N/A

Note that full information on the approval of the study protocol must also be provided in the manuscript.

Field-specific reporting

Please select the one below that is the best fit for your research. If you are not sure, read the appropriate sections before making your selection.

☐ Life sciences ☐ Behavioural & social sciences ☒ Ecological, evolutionary & environmental sciences

For a reference copy of the document with all sections, see nature.com/documents/nr-reporting-summary-flat.pdf

Ecological, evolutionary & environmental sciences study design

All studies must disclose on these points even when the disclosure is negative.

Study description	We apply Gaussian Process modelling in combination with Bayesian inference on a large-scale stable isotope ratio and trace element data set, consisting of 929 timber samples of four genera across 12 Eastern European Countries. Specifically, we (1) combine trace element (TE) and stable isotope ratio (SIR) data obtained from wood samples to perform verification and determination; (2) develop a statistical test to verify a sample against a claimed origin on different spatial scales, taking into account spatial dependency; (3) take a probabilistic approach to spatial modelling of SIR and TE data using Gaussian Processes in order to determine harvest location; (4) incorporate novel prior information based on genus distribution; and, (5) determine which SIR and TE are most important for determining location.
Research sample	905 pith-to-bark wood samples from 11 countries (Belarus, Croatia, Estonia, Finland, Hungary, Latvia, Lithuania, Moldova, Romania, Slovakia, Ukraine) were collected. In addition, existing data from Russian samples was procured through Agroisolab GmbH. These samples were analysed by stable isotope ratio analyis and trace element analysis. The following species were targetted: Betula pendula, Betula pubescens, Betula sp., Fagus sylvatica, Pinus nigra, Pinus sp., Pinus sylvestris, Quercus petraea, Quercus pubescens and Quercus robur.
Sampling strategy	We consider a larger study area and sampling strategy, allowing to capture more climatic variability. For each species, within each country, three trees were sampled within an area of 50 km, the next set of three trees was then 100 to 250 km away, keeping in mind the species distribution. An analysis subset was chosen based on priority genera for timber traceability in Eastern Europe (Betula, Fagus, Pinus and Quercus). Our sampling strategy was aimed at covering entire regions, compared to a "site" based approach.
Data collection	The wood samples were collected by Preferred By Nature from June 2022 until December 2022, following the World Forest ID collection protocol. Metadata (GPS points...) were collected automatically in the World Forest ID collection mobile app. All metadata was checked by curators at the Royal Botanic Gardens, Kew.
Timing and spatial scale	Samples were collected by Preferred by Nature in the following countries: Belarus, Croatia, Estonia, Finland, Hungary, Latvia, Lithuania, Moldova, Romania, Slovakia, Ukraine. Sample collection started in June 2022 until December 2022. The following lists the collection timeframes (year 2022) per country: Lithuania (14 June to 24 August), Romania (7 July to 28 September), Slovakia (9 July to 24 September), Hungary (16 July to 3 September), Finland (21 July until 29 August), Belarus (21 September to 1 December), Croatia (2 August to 11 August), Ukraine (24 August to 27 October), Estonia (9 August to 23 September), Moldova (6 August to 25 August), Latvia (12 August to 24 December). As mentioned above, samples were collected to cover entire regions/countries to maximize our spatial approach (see manuscript).
Data exclusions	There were more samples collected than there are included in the manuscript. This is due to limited analysis budget, however we prioritized the samples which were most important for enforcement questions following the Ukraine invasion. These samples belonged to the following genera: Betula, Fagus, Pinus and Quercus.
Reproducibility	The chemical measurements were performed by the ISO-accredited Agroisolab GmbH in Jülich, Germany. Agroisolab takes part in quarterly ring testing to ensure the reproducibility of their measurements.

Randomization	The data was randomly split into train and test samples to evaluate the accuracy of the method.
Blinding	Blinding was not relevant to this study.

Did the study involve field work? ☒ Yes ☐ No

Field work, collection and transport

Field conditions	N/A
Location	See above (timing and spatial scale)
Access & import/export	All samples were collected in accordance with national law.
Disturbance	N/A

Reporting for specific materials, systems and methods

We require information from authors about some types of materials, experimental systems and methods used in many studies. Here, indicate whether each material, system or method listed is relevant to your study. If you are not sure if a list item applies to your research, read the appropriate section before selecting a response.

Materials & experimental systems

n/a	Involved in the study	
☒	☐	Antibodies
☒	☐	Eukaryotic cell lines
☒	☐	Palaeontology and archaeology
☒	☐	Animals and other organisms
☒	☐	Clinical data
☒	☐	Dual use research of concern
☐	☒	Plants

Methods

n/a	Involved in the study	
☒	☐	ChIP-seq
☒	☐	Flow cytometry
☒	☐	MRI-based neuroimaging

Dual use research of concern

Policy information about dual use research of concern

Hazards

Could the accidental, deliberate or reckless misuse of agents or technologies generated in the work, or the application of information presented in the manuscript, pose a threat to:

No	Yes	
☒	☐	Public health
☒	☐	National security
☒	☐	Crops and/or livestock
☒	☐	Ecosystems
☐	☒	Any other significant area

Other impacts	Safety and security
Hazards	This project was set up as a response to the Ukraine invasion by Russia. To safeguard our collectors, and to prevent the data and associated information to fall into malicious hands, we opt to keep our data and code available upon request. This will allow us to ensure that this information is only available to good actors.

For examples of agents subject to oversight, see the United States Government Policy for Institutional Oversight of Life Sciences Dual Use Research of Concern.

Experiments of concern

Does the work involve any of these experiments of concern:

No	Yes	
☒	☐	Demonstrate how to render a vaccine ineffective
☒	☐	Confer resistance to therapeutically useful antibiotics or antiviral agents
☒	☐	Enhance the virulence of a pathogen or render a nonpathogen virulent
☒	☐	Increase transmissibility of a pathogen
☒	☐	Alter the host range of a pathogen
☒	☐	Enable evasion of diagnostic/detection modalities
☒	☐	Enable the weaponization of a biological agent or toxin
☒	☐	Any other potentially harmful combination of experiments and agents

Precautions and benefits

Biosecurity precautions	*Describe the precautions that were taken during the design and conduct of this research, or will be required in the communication and application of the research, to minimise biosecurity risks. These may include bio-containment facilities, changes to the study design/ methodology or redaction of details from the manuscript.*
Biosecurity oversight	*Describe any evaluations and oversight of biosecurity risks of this work that you have received from people or organizations outside of your immediate team.*
Benefits	*Describe the benefits that application or use of this work could bring, including benefits that may mitigate risks to public health, national security, or the health of crops, livestock or the environment.*
Communication benefits	*Describe whether the benefits of communicating this information outweigh the risks, and if so, how.*

Plants

Seed stocks	As indicated above, wood samples were collected by Preferred by Nature from June 2022 until December 2022. Samples were collected in the following countries: Belarus, Croatia, Estonia, Finland, Hungary, Latvia, Lithuania, Moldova, Romania, Slovakia, Ukraine. As samples were collected across regions/countries, we refrain from listing all individual locations. The locations can be seen in our manuscript. These samples are now part of the World Forest ID Georeferenced Sample Collection at the Royal Botanic Gardens, Kew.
Novel plant genotypes	*Describe the methods by which all novel plant genotypes were produced. This includes those generated by transgenic approaches, gene editing, chemical/radiation-based mutagenesis and hybridization. For transgenic lines, describe the transformation method, the number of independent lines analyzed and the generation upon which experiments were performed. For gene-edited lines, describe the editor used, the endogenous sequence targeted for editing, the targeting guide RNA sequence (if applicable) and how the editor was applied.*
Authentication	*Describe any authentication procedures for each seed stock used or novel genotype generated. Describe any experiments used to assess the effect of a mutation and, where applicable, how potential secondary effects (e.g. second site T-DNA insertions, mosiacism, off-target gene editing) were examined.*
